# The genome sequence of the lesser stag beetle, *Dorcus parallelipipedus *(Linnaeus, 1758)

**DOI:** 10.12688/wellcomeopenres.21262.1

**Published:** 2024-04-17

**Authors:** Liam M. Crowley, Dominic Phillips, University of Oxford and Wytham Woods Genome Acquisition Lab, Darwin Tree of Life Barcoding collective, Wellcome Sanger Institute Tree of Life Management, Samples and Laboratory team, Wellcome Sanger Institute Scientific Operations: Sequencing Operations, Wellcome Sanger Institute Tree of Life Core Informatics team, Tree of Life Core Informatics collective, Darwin Tree of Life Consortium

**Affiliations:** 1 University of Oxford, Oxford, England, UK; 2 Natural History Museum, London, England, UK

**Keywords:** Dorcus parallelipipedus, lesser stag beetle, genome sequence, chromosomal, Coleoptera

## Abstract

We present a genome assembly from an individual male *Dorcus parallelipipedus* (the lesser stag beetle; Arthropoda; Insecta; Coleoptera; Lucanidae). The genome sequence is 470.9 megabases in span. Most of the assembly is scaffolded into 10 chromosomal pseudomolecules, including the X and Y sex chromosomes. The mitochondrial genome has also been assembled and is 18.19 kilobases in length.

## Species taxonomy

Eukaryota; Opisthokonta; Metazoa; Eumetazoa; Bilateria; Protostomia; Ecdysozoa; Panarthropoda; Arthropoda; Mandibulata; Pancrustacea; Hexapoda; Insecta; Dicondylia; Pterygota; Neoptera; Endopterygota; Coleoptera; Polyphaga; Scarabaeiformia; Scarabaeoidea; Lucanidae; Lucaninae; *Dorcus*; *Dorcus parallelipipedus* (Linnaeus, 1758) (NCBI:txid41107).

## Background

*Dorcus parallelipipedus* (Linnaeus, 1758), also known as the Lesser Stag Beetle, is a species of beetle in the Lucanidae family, commonly referred to as the Stag Beetles. *D. parallelipipedus* is the only member of its genus in the UK and can be distinguished from members of the closely related genus *Lucanus* via the presence of a sharp medioexternal tooth on the hind and mid tibiae, black coloured upperside and striate fore tibial sculpture ( Duff & Schmidt, 2020). This species may also be distinguished by an enlarged 7th antennomere, 3 segmented antennal club and a large mediointernal tooth on the mandible ( Duff & Schmidt, 2020). Females possess a pair of median tubercles on the frons, the pronotum is as wide as the elytra and the entire body is shiny and punctured ( Duff & Schmidt, 2020; UK Beetles, 2024). Among UK beetles, *D. parallelipipedus* is easily identified due to its large size, measuring between 20–32 mm ( UK Beetles, 2024).

Adult *Dorcus parallelipipedus* can be found throughout the year. During winter months, they inhabit soft wood or piled vegetation. From April to September, particularly in spring and summer, they exhibit activity both during the day and at night ( Duff & Schmidt, 2020). They are proficient fliers and are attracted to light sources. These beetles have a diverse array of host trees, including oak, lime, elder, willow, elm, beech, and various fruit trees ( UK Beetles, 2024). The adult life stage can span several years and they may cohabit with larvae in wood. Females create small depressions or short tunnels in wood or bark before laying a single egg. The larval phase can last up to three years, with larvae occasionally congregating in heavily consumed wood pieces. Pupation typically occurs in summer or autumn within a chamber prepared by the larva, usually just beneath the bark. Adults emerge in late summer or autumn, when they feed primarily on sap, and they are known to be drawn to substances like syrup, treacle and ginger ( UK Beetles, 2024).

*Dorcus parallelipipedus* is globally distributed throughout Europe, from Portugal through to Russia, going as far north to southern Sweden. It has also been recorded through Anatolia and Israel ( UK Beetles, 2024). Within the UK, *D. parallelipipedus* occurs throughout England, with few records more northwards than Nottinghamshire, being seemingly absent from Cornwall, West Wales and Scotland ( NBN Atlas Partnership, 2024). Though it appears to be common throughout its range, it has suffered recent declines throughout its full range – much like other saproxylic beetles.

The whole mitochondrial genome of *Dorcus parallelipipedus* was sequenced by Linard *et al*. (2016) and later used in a phylogenetic analysis by Chen *et al*. (2018) to investigate the relationships between two new complete mitochondrial genomes of other *Dorcus* stag beetles. The full genome of *D. parallelipipedus* generated by the Darwin Tree of Life aims to complement this previous research on the mitochondrial genomes of this species and its relatives. Though registered as Least concern on the IUCN red list ( Thomaes *et al.*, 2015), the continued decline of other saproxylic beetles ( Hagge *et al.*, 2024; Sikora *et al.*, 2023) highlights the importance of studying the full genome of these species and how we can use this information to assist in the conservation of such important species.

The genome of *Dorcus parallelipipedus* was sequenced as part of the Darwin Tree of Life Project, a collaborative effort to sequence all named eukaryotic species in the Atlantic Archipelago of Britain and Ireland. Here we present a chromosomally complete genome sequence for *Dorcus parallelipipedus*, based on one specimen collected from Wytham Woods, Oxfordshire.

## Genome sequence report

**Figure 1.**
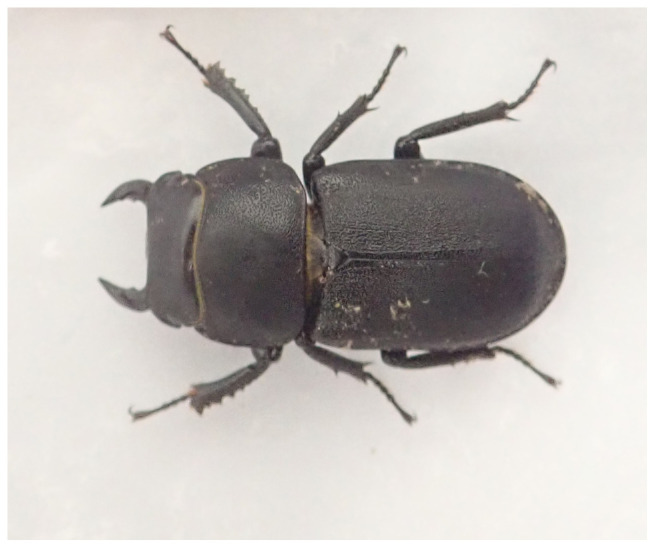
Photograph of the *Dorcus parallelipipedus* (icDorPara1) specimen used for genome sequencing.

The genome was sequenced from one male *Dorcus parallelipipedus* ( Figure 1) collected from Wytham Woods, Oxfordshire, UK (51.77, –1.34). A total of 38-fold coverage in Pacific Biosciences single-molecule HiFi long reads was generated. Primary assembly contigs were scaffolded with chromosome conformation Hi-C data. Manual assembly curation corrected 101 missing joins or mis-joins and removed 19 haplotypic duplications, reducing the assembly length by 0.57% and the scaffold number by 42.21%, and increasing the scaffold N50 by 3.36%.

**Figure 2.**
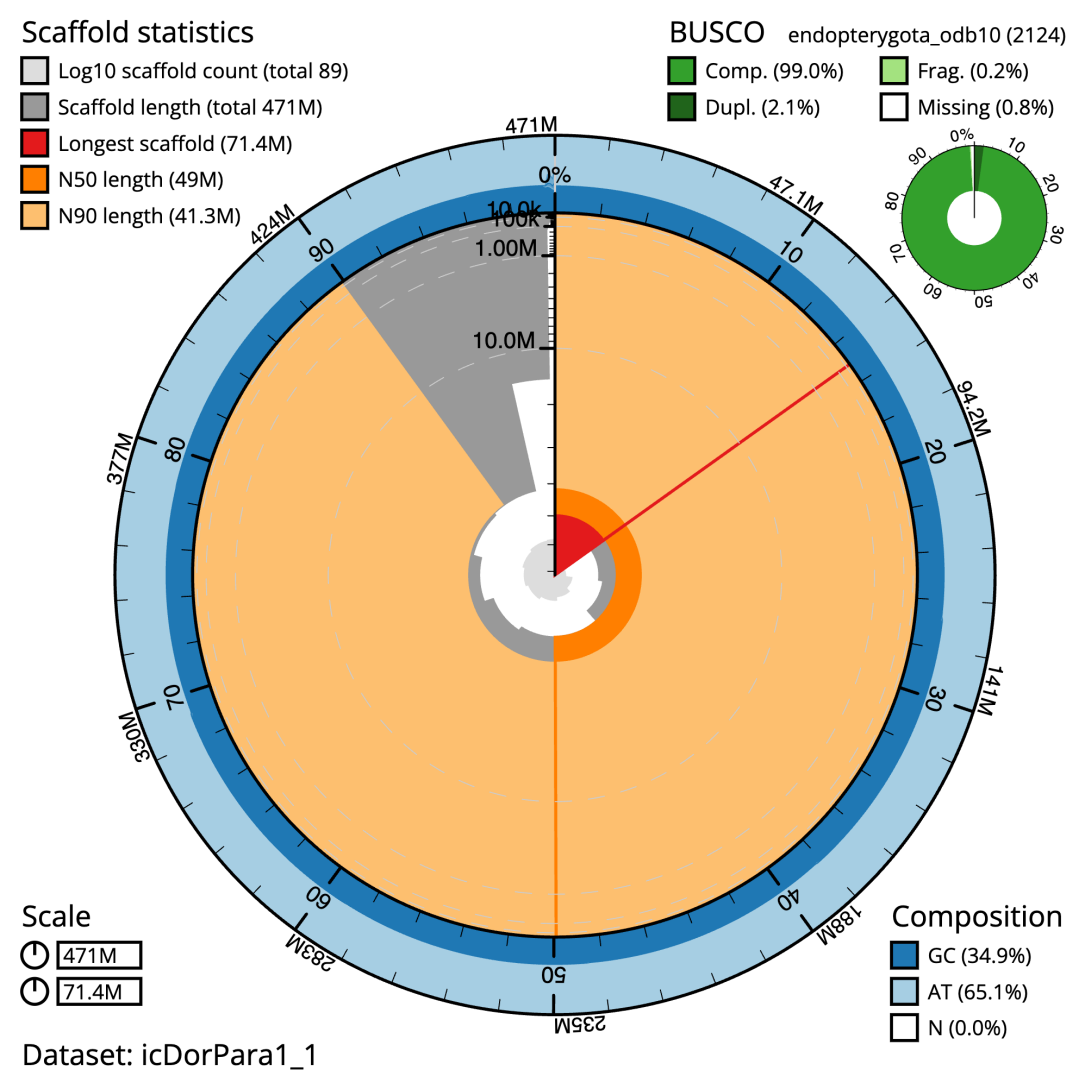
Genome assembly of *Dorcus parallelipipedus*, icDorPara1.1: metrics. The BlobToolKit snail plot shows N50 metrics and BUSCO gene completeness. The main plot is divided into 1,000 size-ordered bins around the circumference with each bin representing 0.1% of the 470,898,720 bp assembly. The distribution of scaffold lengths is shown in dark grey with the plot radius scaled to the longest scaffold present in the assembly (71,441,279 bp, shown in red). Orange and pale-orange arcs show the N50 and N90 scaffold lengths (48,965,044 and 41,300,529 bp), respectively. The pale grey spiral shows the cumulative scaffold count on a log scale with white scale lines showing successive orders of magnitude. The blue and pale-blue area around the outside of the plot shows the distribution of GC, AT and N percentages in the same bins as the inner plot. A summary of complete, fragmented, duplicated and missing BUSCO genes in the endopterygota_odb10 set is shown in the top right. An interactive version of this figure is available at https://blobtoolkit.genomehubs.org/view/icDorPara1_1/dataset/icDorPara1_1/snail.

**Figure 3.**
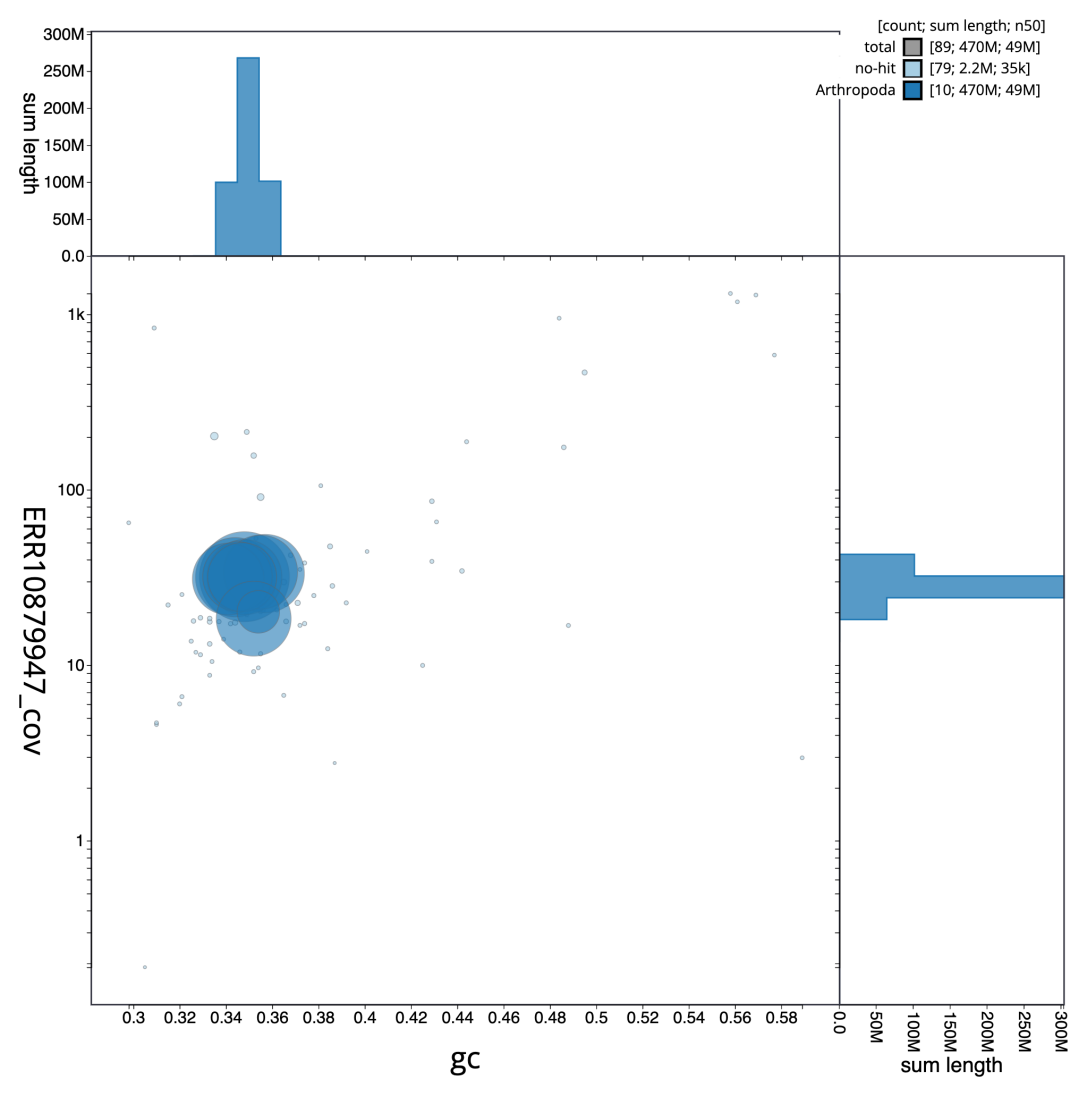
Genome assembly of *Dorcus parallelipipedus*, icDorPara1.1: BlobToolKit GC-coverage plot. Sequences are coloured by phylum. Circles are sized in proportion to sequence length. Histograms show the distribution of sequence length sum along each axis. An interactive version of this figure is available at https://blobtoolkit.genomehubs.org/view/icDorPara1_1/dataset/icDorPara1_1/blob.

**Figure 4.**
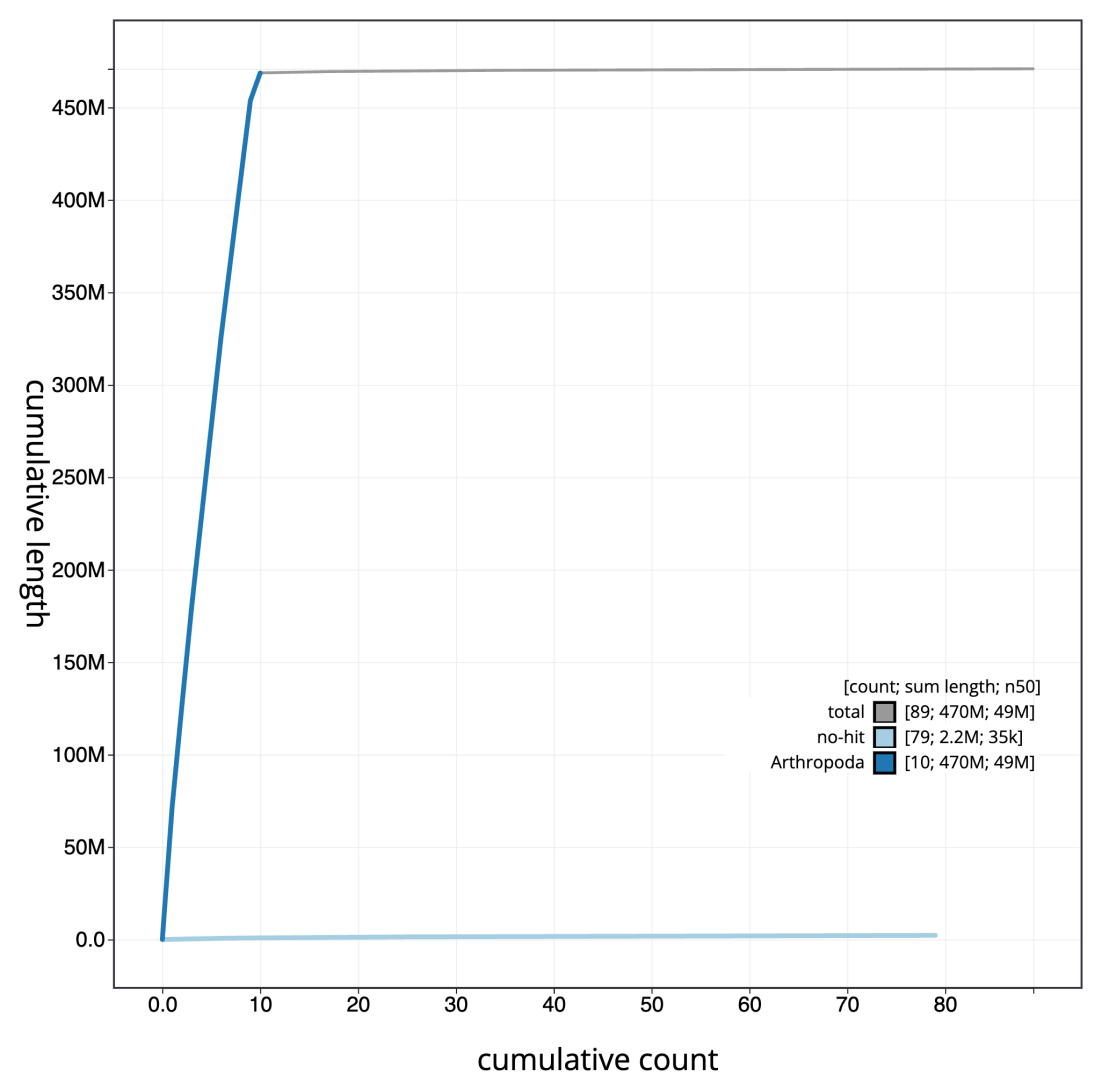
Genome assembly of *Dorcus parallelipipedus*, icDorPara1.1: BlobToolKit cumulative sequence plot. The grey line shows cumulative length for all sequences. Coloured lines show cumulative lengths of sequences assigned to each phylum using the buscogenes taxrule. An interactive version of this figure is available at https://blobtoolkit.genomehubs.org/view/icDorPara1_1/dataset/icDorPara1_1/cumulative.

**Figure 5.**
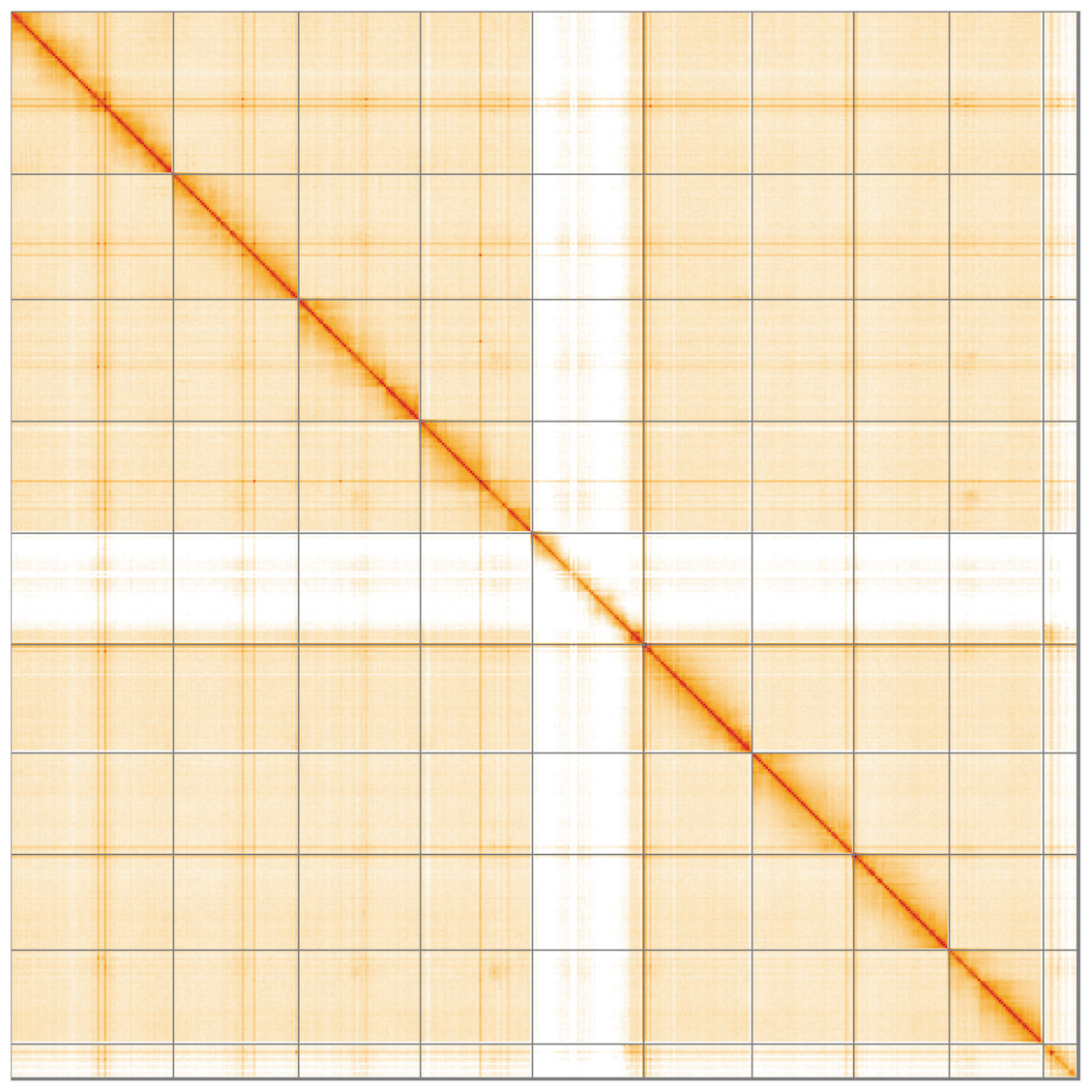
Genome assembly of *Dorcus parallelipipedus*, icDorPara1.1: Hi-C contact map of the icDorPara1.1 assembly, visualised using HiGlass. Chromosomes are shown in order of size from left to right and top to bottom. An interactive version of this figure may be viewed at https://genome-note-higlass.tol.sanger.ac.uk/l/?d=H-frUEYlQDGBaKkAm-ppPg.

The final assembly has a total length of 470.9 Mb in 88 sequence scaffolds with a scaffold N50 of 49.0 Mb ( Table 1). The snail plot in Figure 2 provides a summary of the assembly statistics, while the distribution of assembly scaffolds on GC proportion and coverage is shown in Figure 3. The cumulative assembly plot in Figure 4 shows curves for subsets of scaffolds assigned to different phyla. Most (99.54%) of the assembly sequence was assigned to 10 chromosomal-level scaffolds, representing 8 autosomes and the X and Y sex chromosomes. Chromosome-scale scaffolds confirmed by the Hi-C data are named in order of size ( Figure 5; Table 2). Chromosomes X and Y were assigned based on read coverage statistics. While not fully phased, the assembly deposited is of one haplotype. Contigs corresponding to the second haplotype have also been deposited. The mitochondrial genome was also assembled and can be found as a contig within the multifasta file of the genome submission.

**Table 1.** Genome data for *Dorcus parallelipipedus*, icDorPara1.1.

Project accession data		
Assembly identifier	icDorPara1.1	
Species	*Dorcus parallelipipedus*	
Specimen	icDorPara1	
NCBI taxonomy ID	41107	
BioProject	PRJEB59788	
BioSample ID	SAMEA7701270	
Isolate information	icDorPara1, male: head (DNA sequencing), thorax (Hi-C sequencing)	
Assembly metrics *		*Benchmark*
Consensus quality (QV)	61.7	*≥ 50*
*k*-mer completeness	100.0%	*≥ 95%*
BUSCO **	C:99.0%[S:96.9%,D:2.1%], F:0.2%,M:0.8%,n:2,124	*C ≥ 95%*
Percentage of assembly mapped to chromosomes	99.54%	*≥ 95%*
Sex chromosomes	XY	*localised homologous pairs*
Organelles	Mitochondrial genome: 18.19 kb	*complete single alleles*
Raw data accessions		
PacificBiosciences SEQUEL II	ERR10879947	
Hi-C Illumina	ERR10890734	
Genome assembly		
Assembly accession	GCA_958336345.1	
*Accession of alternate haplotype*	GCA_958336325.1	
Span (Mb)	470.9	
Number of contigs	763	
Contig N50 length (Mb)	1.4	
Number of scaffolds	88	
Scaffold N50 length (Mb)	49.0	
Longest scaffold (Mb)	71.44	

* Assembly metric benchmarks are adapted from column VGP-2020 of “Table 1: Proposed standards and metrics for defining genome assembly quality” from Rhie *et al.* (2021). ** BUSCO scores based on the endopterygota_odb10 BUSCO set using version 5.3.2. C = complete [S = single copy, D = duplicated], F = fragmented, M = missing, n = number of orthologues in comparison. A full set of BUSCO scores is available at https://blobtoolkit.genomehubs.org/view/icDorPara1_1/dataset/icDorPara1_1/busco.

**Table 2.** Chromosomal pseudomolecules in the genome assembly of *Dorcus parallelipipedus*, icDorPara1.

INSDC accession	Chromosome	Length (Mb)	GC%
OY284475.1	1	71.44	35.0
OY284476.1	2	55.04	34.5
OY284477.1	3	53.55	35.5
OY284478.1	4	49.19	35.0
OY284480.1	5	47.61	35.5
OY284481.1	6	44.66	34.0
OY284482.1	7	42.01	34.5
OY284483.1	8	41.3	34.5
OY284479.1	X	48.97	35.0
OY284484.1	Y	14.91	35.5
OY284485.1	MT	0.02	31.0

The estimated Quality Value (QV) of the final assembly is 61.7 with *k*-mer completeness of 100.0%, and the assembly has a BUSCO v5.3.2 completeness of 99.0% (single = 96.9%, duplicated = 2.1%), using the endopterygota_odb10 reference set ( *n* = 2,124).

Metadata for specimens, barcode results, spectra estimates, sequencing runs, contaminants and pre-curation assembly statistics are given at https://links.tol.sanger.ac.uk/species/41107.

## Methods

### Sample acquisition and nucleic acid extraction

A male *Dorcus parallelipipedus* (specimen ID Ox000491, ToLID icDorPara1) was collected from Wytham Woods, Oxfordshire (biological vice-county Berkshire), UK (latitude 51.77, longitude –1.34) on 2020-06-20 by potting. The specimen was collected and identified by Liam Crowley (University of Oxford) and preserved on dry ice.

The workflow for high molecular weight (HMW) DNA extraction at the Wellcome Sanger Institute (WSI) includes a sequence of core procedures: sample preparation; sample homogenisation, DNA extraction, fragmentation, and clean-up. In sample preparation, the icDorPara1 sample was weighed and dissected on dry ice ( Jay *et al.*, 2023). Tissue from the head was homogenised using a PowerMasher II tissue disruptor ( Denton *et al.*, 2023a). HMW DNA was extracted using the Automated MagAttract v1 protocol ( Sheerin *et al.*, 2023). DNA was sheared into an average fragment size of 12–20 kb in a Megaruptor 3 system with speed setting 30 ( Todorovic *et al.*, 2023). Sheared DNA was purified by solid-phase reversible immobilisation ( Strickland *et al.*, 2023): in brief, the method employs a 1.8X ratio of AMPure PB beads to sample to eliminate shorter fragments and concentrate the DNA. The concentration of the sheared and purified DNA was assessed using a Nanodrop spectrophotometer and Qubit Fluorometer and Qubit dsDNA High Sensitivity Assay kit. Fragment size distribution was evaluated by running the sample on the FemtoPulse system.

Protocols developed by the WSI Tree of Life laboratory are publicly available on protocols.io ( Denton *et al.*, 2023b).

### Sequencing

Pacific Biosciences HiFi circular consensus DNA sequencing libraries were constructed according to the manufacturers’ instructions. DNA sequencing was performed by the Scientific Operations core at the WSI on a Pacific Biosciences SEQUEL II instrument. Hi-C data were also generated from thorax tissue of icDorPara1 using the Arima2 kit and sequenced on the Illumina NovaSeq 6000 instrument.

### Genome assembly, curation and evaluation

Assembly was carried out with Hifiasm ( Cheng *et al.*, 2021) and haplotypic duplication was identified and removed with purge_dups ( Guan *et al.*, 2020). The assembly was then scaffolded with Hi-C data ( Rao *et al.*, 2014) using YaHS ( Zhou *et al.*, 2023). The assembly was checked for contamination and corrected using the TreeVal pipeline ( Pointon *et al.*, 2023). Manual curation was performed using JBrowse2 ( Diesh *et al.*, 2023), HiGlass ( Kerpedjiev *et al.*, 2018) and PretextView ( Harry, 2022). The mitochondrial genome was assembled using MitoHiFi ( Uliano-Silva *et al.*, 2023), which runs MitoFinder ( Allio *et al.*, 2020) or MITOS ( Bernt *et al.*, 2013) and uses these annotations to select the final mitochondrial contig and to ensure the general quality of the sequence.

A Hi-C map for the final assembly was produced using bwa-mem2 ( Vasimuddin *et al.*, 2019) in the Cooler file format ( Abdennur & Mirny, 2020). To assess the assembly metrics, the *k*-mer completeness and QV consensus quality values were calculated in Merqury ( Rhie *et al.*, 2020). This work was done using Nextflow ( Di Tommaso *et al.*, 2017) DSL2 pipelines “sanger-tol/readmapping” ( Surana *et al.*, 2023a) and “sanger-tol/genomenote” ( Surana *et al.*, 2023b). The genome was analysed within the BlobToolKit environment ( Challis *et al.*, 2020) and BUSCO scores ( Manni *et al.*, 2021; Simão *et al.*, 2015) were calculated.

Table 3 contains a list of relevant software tool versions and sources.

**Table 3.** Software tools: versions and sources.

Software tool	Version	Source
BlobToolKit	4.1.7	https://github.com/blobtoolkit/blobtoolkit
BUSCO	5.3.2	https://gitlab.com/ezlab/busco
Hifiasm	0.16.1-r375	https://github.com/chhylp123/hifiasm
HiGlass	1.11.6	https://github.com/higlass/higlass
Merqury	MerquryFK	https://github.com/thegenemyers/MERQURY.FK
MitoHiFi	2	https://github.com/marcelauliano/MitoHiFi
PretextView	0.2	https://github.com/wtsi-hpag/PretextView
purge_dups	1.2.3	https://github.com/dfguan/purge_dups
sanger-tol/genomenote	v1.0	https://github.com/sanger-tol/genomenote
sanger-tol/readmapping	1.1.0	https://github.com/sanger-tol/readmapping/tree/1.1.0
TreeVal	-	https://github.com/sanger-tol/treeval
YaHS	yahs-1.1.91eebc2	https://github.com/c-zhou/yahs

### Wellcome Sanger Institute – Legal and Governance

The materials that have contributed to this genome note have been supplied by a Darwin Tree of Life Partner. The submission of materials by a Darwin Tree of Life Partner is subject to the **‘Darwin Tree of Life Project Sampling Code of Practice’**, which can be found in full on the Darwin Tree of Life website here. By agreeing with and signing up to the Sampling Code of Practice, the Darwin Tree of Life Partner agrees they will meet the legal and ethical requirements and standards set out within this document in respect of all samples acquired for, and supplied to, the Darwin Tree of Life Project.

Further, the Wellcome Sanger Institute employs a process whereby due diligence is carried out proportionate to the nature of the materials themselves, and the circumstances under which they have been/are to be collected and provided for use. The purpose of this is to address and mitigate any potential legal and/or ethical implications of receipt and use of the materials as part of the research project, and to ensure that in doing so we align with best practice wherever possible. The overarching areas of consideration are:

•   Ethical review of provenance and sourcing of the material

•   Legality of collection, transfer and use (national and international)

Each transfer of samples is further undertaken according to a Research Collaboration Agreement or Material Transfer Agreement entered into by the Darwin Tree of Life Partner, Genome Research Limited (operating as the Wellcome Sanger Institute), and in some circumstances other Darwin Tree of Life collaborators.

## Data Availability

European Nucleotide Archive: *Dorcus parallelipipedus* (lesser stag beetle). Accession number PRJEB59788; https://identifiers.org/ena.embl/PRJEB59788 ( Wellcome Sanger Institute, 2023). The genome sequence is released openly for reuse. The *Dorcus parallelipipedus* genome sequencing initiative is part of the Darwin Tree of Life (DToL) project. All raw sequence data and the assembly have been deposited in INSDC databases. The genome will be annotated using available RNA-Seq data and presented through the Ensembl pipeline at the European Bioinformatics Institute. Raw data and assembly accession identifiers are reported in Table 1. Members of the University of Oxford and Wytham Woods Genome Acquisition Lab are listed here: https://doi.org/10.5281/zenodo.7125292. Members of the Darwin Tree of Life Barcoding collective are listed here: https://doi.org/10.5281/zenodo.4893703. Members of the Wellcome Sanger Institute Tree of Life Management, Samples and Laboratory team are listed here: https://doi.org/10.5281/zenodo.10066175. Members of Wellcome Sanger Institute Scientific Operations: Sequencing Operations are listed here: https://doi.org/10.5281/zenodo.10043364. Members of the Wellcome Sanger Institute Tree of Life Core Informatics team are listed here: https://doi.org/10.5281/zenodo.10066637. Members of the Tree of Life Core Informatics collective are listed here: https://doi.org/10.5281/zenodo.5013541. Members of the Darwin Tree of Life Consortium are listed here: https://doi.org/10.5281/zenodo.4783558.
