# Supplementary material for: The genome sequence of the lesser stag beetle, Dorcus parallelipipedus (Linnaeus, 1758)
Source: Wellcome Open Res. 2024 Apr 17;9:202. [Version 1] doi: 10.12688/wellcomeopenres.21262.1 (PMC11342025; doi:10.12688/wellcomeopenres.21262.1)
Supplement: Peer review history

# Reviewer report: Reviewer response for version 1

**DOI:** 10.21956/wellcomeopenres.23516.r91308

**Published:** 2024-09-17

**Authors:** Terrence Sylvester

**Affiliations:** 1 The University of Memphis, Memphis, Tennessee, USA

The authors present the genome sequence of the lesser stag beetle. The authors have done an excellent job of generating the genome assembly and scaffolding it up to chromosome level. The present genome could further benefit from including an annotation. The authors follow a standard and widely accepted genome sequencing and assembly pipeline. Data are well presented and publicly available. I do not have other significant remarks on the work presented in this manuscript.

Are sufficient details of methods and materials provided to allow replication by others?

Yes

Is the rationale for creating the dataset(s) clearly described?

Yes

Are the datasets clearly presented in a useable and accessible format?

Yes

Are the protocols appropriate and is the work technically sound?

Yes

Reviewer Expertise:

Genomics

I confirm that I have read this submission and believe that I have an appropriate level of expertise to confirm that it is of an acceptable scientific standard.

# Reviewer report: Reviewer response for version 1

**DOI:** 10.21956/wellcomeopenres.23516.r92713

**Published:** 2024-08-22

**Authors:** Robert Angus

**Affiliations:** 1 The Natural History Museum, London, UK

This is a clearly presented account of the genome of *Dorcus parallelipipedus. *The methods used are standard and the illustration of the beetle leaves no doubt of its identity.

Are sufficient details of methods and materials provided to allow replication by others?

Yes

Is the rationale for creating the dataset(s) clearly described?

Yes

Are the datasets clearly presented in a useable and accessible format?

Yes

Are the protocols appropriate and is the work technically sound?

Yes

Reviewer Expertise:

NA

I confirm that I have read this submission and believe that I have an appropriate level of expertise to confirm that it is of an acceptable scientific standard.

# Reviewer report: Reviewer response for version 1

**DOI:** 10.21956/wellcomeopenres.23516.r89041

**Published:** 2024-08-09

**Authors:** Themistoklis Giannoulis

**Affiliations:** 1 University of Thessaly, Larissa, Greece

The article is very well written, it described the goal clearly and the methods are very well presented. It follows the format of other articles, which aim towards the same goal "One Species - One Genome". The authors have used cutting-edge technologies of DNA sequencing and state-of-the-art algorithms to analyze and present the data.

Are sufficient details of methods and materials provided to allow replication by others?

Yes

Is the rationale for creating the dataset(s) clearly described?

Yes

Are the datasets clearly presented in a useable and accessible format?

Yes

Are the protocols appropriate and is the work technically sound?

Yes

Reviewer Expertise:

Animal Genetics and Genomics, Phylogenetics, Evolution

I confirm that I have read this submission and believe that I have an appropriate level of expertise to confirm that it is of an acceptable scientific standard.
